# Notes and Comments

**Published:** 1895-09

**Authors:** 


### Notes and Comments.[1]

The Dentist's Duties to Himself and his Clients.—Since the first issue of the *Dental Digest*, Dr. J. N. Crouse has contributed to that journal a series of practical papers upon conducting a dental practice upon business principles. In the May number there are some suggestions which every young man should read. Dr. Crouse asks, "Have you always gone to your chair or to the work in hand in the best physical condition possible?" This is a matter of importance; if the hand is unsteady and the eye dimmed, our place is not at the chair. We should avoid excesses, and see to it that our diet is correct, our sleep full and refreshing, so that with a clear head we can "think and reason correctly, and carry out well-laid plans with skill and dexterity."

One of the most pertinent questions asked is, "Do we treat our patients as if they were living beings, worthy of the most kindly care?" It is owing to the neglect of this one point that there exists so much dread of the dental chair. Too often does the operator forget or seem to be indifferent to the fact that he is working upon *living beings* with all the sensibilities he himself possesses.

System of Charging for Dental Services.—In the article referred to above Dr. Crouse makes a plea for all practitioners to

[1] The assistant editor solicits contributions for this department,—new methods, new remedies and formulas, or any short practical note which may prove of value to the practitioner or student. Address 1718 Walnut Street, Philadelphia.

adopt the system of charging by the hour for their services, and not according to the amount of work done. This, to the writer, has always seemed to be a good plan, where possible to carry out, but to expect it to be universally adopted is out of the question; in fact, there are a very few who can do it. The doctor thinks it places temptation out of the way. That is, if it would be better practice to cut down a thin wall between two cavities in the masticating surface of a molar tooth, for instance, this would be done, and one large filling made of it instead of two small ones, to make a better showing upon the bill of items. Now the latter—the bill of *items*—is a much worse practice than making our charges according to the services rendered. The writer has but once done this (then it was requested), as he considers it a step towards retrogression. If a man does not wish to be strictly honest in his dealings, the adoption of this system of charging will not make him so. Our rule, both in deciding upon the practice to pursue and in making charges for the same, has always been the "Golden Rule," laid down for us centuries ago.

---

Teacher and Taught.—In writing upon this subject, John B. Roberts, M.D., truly says that the faculty of imparting knowledge is distinct from the faculty of acquiring knowledge. To teach is not simply to tell, but to make the statement of fact so interesting and so clear that it assumes a living importance, and is eagerly sought and intelligently retained by the hearer as a part of himself. A teacher must draw bold, clear outlines, omitting details, and repeating essentials until his pupils have a mental framework upon which they themselves may erect more elaborate structures at a future time. He who has not the power to select the essentials and lead the scholar to reason and observe is destitute of the teaching instinct. Then his lectures become mere recitations, as wearisome to himself as to his involuntary hearers. The true teacher furnishes his pupil with compass and chart, no more. The latter must select his route and reach his harbor by the exercise of those intellectual powers which have been given him. It is experience and not memory that has been called the mother of ideas.

